# The Nursing Supplement

**Published:** 1889-11-23

**Authors:** 


*The Hospital,*
NOVEMBER 23, 1889.

### The Nursing Mirror.

*Extra Supplement*

All Contributions for this Supplement should be addressed to the Editor, THE HOSPITAL, 139-140, Salisbury Court, Fleet Street, London, E.C., and should have the word "Nursing" plainly written in left-hand top corner of the envelope.

### En Passant.

INTOLERANCE.—As we grow older most of us become more tolerant, more lenient towards the faulty, less severe in our judgments of others. This is especially the case amongst nurses, who should ever strive to find some good amongst things evil, and to have tenderness and compassion for all. And that toleration given to others should also be theirs. How then is it we find the following statement in a contemporary? "Nurse Constance, of the Trained Nurses' Institute, Canterbury, has been dismissed because on October 18th she was received into the Roman Church. As the institute solicits contributions on the ground of its non-sectarian character the action of the committee requires explanation."

THE PRACTICAL ASYLUMS BOARD.—We all know how the nursing and nurses of the Metropolitan Asylums Board hospitals have lately improved, and we gave, a fortnight ago, the views of the board on the advantage of trained matrons. Now we find in the report the following practical remarks on sisters' dresses: "The question of the substitution of serge uniform dresses in lieu of gingham for day and night superintendent nurses at all the infectious hospitals, has been considered by your committee pursuant to reference from the board of the 21st September last. Your committee do not think that the substitution of serge is desirable, (1) because the material would rapidly deteriorate in warmth and in texture by the frequent washing, which its constant exposure to infection would necessitate; and (2) because it appears to your committee very undesirable that woollen materials, which absorb and give out dust and other particles so easily, should be worn in an infectious ward—at least, as external garments. Your committee, therefore, recommend that a stout jean be substituted for gingham, and that the Contract Committee be instructed accordingly."

TAUNTON INSTITUTION.—The following is the first annual report of the Victoria Jubilee Nursing Institution at Taunton: "In accordance with the proposition contained in the rules for the institute, five nurses have been appointed ward nurses, at a salary of £25 a year each, and a code of instructions for their guidance has been drawn up. The staff of nurses on the 6th November, 1888, consisted of 16; of these four have since resigned. The applications of numerous candidates for election as pupil nurses have been considered: 19 were accepted on probation. Of these seven retired, 11 were elected on the staff, and one has not yet completed her probation. The staff of nurses now numbers 23. The lady superintendent of nurses and some of the senior nurses have received instructions in massage. Classes for instruction of nurses in elementary anatomy and physiology are being given by the house surgeon, and in general nursing by the lady superintendent of nurses. A scheme for nursing the sick poor of the town, submitted by Dr. Liddon, has been under the consideration of the committee, who regret that they are not at present in a position to carry out so large an undertaking. Many applications have been received to supply nurses, but owing to the number of our efficient nurses being limited, the demand has hitherto been only very partially met. The thanks of the committee are due to Miss Portman for undertaking to procure books for a library for the nurses."

NURSING IN THE NORTH.—A meeting has been held at Dundee in aid of a district nursing society. Miss Guthrie Wright, the superintendent of the Edinburgh Nursing Home, the headquarters of the Scottish branch of the Queen Victoria Jubilee Nurses' Institute, explained the character and scope of the work very clearly in a lengthy address. Lord Strathmore moved the following resolution: "That this meeting, being convinced that the condition of the sick poor of the city can be greatly ameliorated by competent nursing, resolves that a society be formed, having for its object the nursing of the sick poor in their own homes by means of thoroughly-trained and qualified nurses, under the name of 'The Dundee Sick Poor Nursing Society.' Also, that the society be affiliated with the Scottish branch of the Queen Victoria Jubilee Institute for Nurses." The meeting was very enthusiastic, and an able committee was appointed to start the work.

NURSING HOME AT BRISTOL.—The new nursing home for twenty-six nurses, which is to be opened on January 1, at Bristol is nearly finished. All the different features of the establishment have been carefully considered, and Dr. Shekleton has personally superintended the undertaking in its different stages. The building will be known as the Bristol Royal Infirmary Nurses' Home and Institute, but, though primarily intended for the accommodation of those in the infirmary, it is also hoped to render it of service to other nurses who may, when occasion requires, be engaged privately. Each nurse will have a room to herself, in which will be placed a gas stove, chest of drawers, etc., so that she may be able to enjoy the convenience and privacy of a home, difficult to do in the wards of the infirmary. The rooms are approached by galleries, which impart an air of lightness to the interior of the institution, the woodwork being of pitch pine in two colours. Great care has been taken with sanitary and ventilating apparatus, and the nurses of Bristol are to be congratulated on their new home.

SHORT ITEMS.—Sisters Erskine, Beresford, and Neville (Indian Army Service) have been thanked for their services during the late epidemic of typhoid.—A hospital surgeon writes to the *Lancet* to know whether nurses' bedrooms should be placed in the roof as attics over surgical wards. Certainly not; what architect can propose or committee accept such an idiotic arrangement?—A new patent bed called the "turnover," the invention of a nurse, was exhibited last week at the Fine Arts Rooms, Gower-street.—Much vexation is felt at Guy's at the reopening of the old question about the nursing there; all is now working harmoniously, and bygones should be bygones.—Five French sisters of mercy have been appointed to the Government Hospital, Hong Kong; English nurses are hurt and disappointed that Government appointments should be given to French sisters for no reason but cheapness.—A lot of nurses were lately hoaxed by letters purporting to come from the Mayor of Bath, who is a medical practitioner.—St. Paul's, Portsmouth, is appealing for funds to start a parish nurse.—At the annual meeting of Longton Cottage Hospital, in answer to Mr. Brough, the matron (Miss Ashdown) explained that the falling off of the receipts of the Harding Nursing Institution was due to the fact that the number of nurses was now very much less than formerly. During the past year the number of nurses had been four part of the time and three the remainder, and the profits realised £171, which she considered was a capital result.

### A Hospital Boy.

"He's a little nuisance!" declared the doctor of the cottage hospital with a poor attempt at a frown. "I believe he spends six months in the year as an inmate here. Confound the boy, he actually seems to like breaking his bones!"

It really seemed like it, for Toby Brown "came up smiling" after accidents such as would have killed off any ordinary boy; in addition to which Toby had caught—and triumphed over—every disease known to childhood. The consequence was that not even the nurses themselves were more at home in the hospital than the ever-reappearing little patient.

"Ee do enjy hisself in 'orsepital, Toby do!" Mrs. Brown would declare, affably implying the highest of compliments to the hospital.

"Well, it's a good thing he likes it," said the matron, "for the poor little man will be a prisoner, this time, for a matter of six or eight weeks—perhaps more."

"Do 'ee say so, ma'am!" The tearful mother seemed half gratified, all the same, that her boy had distinguished himself by a more serious accident than usual. "What do the doctor say as it is?"

"His thigh is fractured."

"There!" interrupted Mrs. Brown. "Toby, you hear the lady! Ain't you 'shamed of yourself a-doin' such a horful thing?"

"Well, but mother, how could I help it?" spoke up a half-frightened voice from the bed on which Toby lay flat on his back; "if so be as *you'd* found a young rook lying on the ground, tumbled out o' his nest, why, you'd ha' climbed the old elm yourself to put he back."

"Did ever anyone hear the like o' that?" said the indignant Mrs. Brown.

"There, there!" put in the matron, pacifically; "it was a kind thought to put back the poor bird, Toby, and we must do our best to make you well. In six weeks or so we'll have you out of bed, and into plaster of Paris, see if we don't."

Meantime, Toby "enjoyed" the comforts he loved so much, and when the time came to be liberated, he was sorry enough to leave the hospital.

"It won't be for long," laughed a nurse, watching his departure. "We shall soon be having him back again." But when Toby was next heard of it was in quite a new light.

"Mother, what *do* you think?" said Johnny, the little boy up at the great house of the village. "You know that little shaver you and Edie and I saw in the hospital lately? Well, this afternoon, when I was coming home from the rectory, I ran into a cottage to ask for a drink of water, and there was little Brown sitting beside a bed in the corner, where a smaller chap was lying ill. You never saw anything so comical as the fellow looked. I said: 'Hilloa! what are *you* doing here?' And he said, as solemn as an owl: 'I be wisitin' the sick, Master Johnny!' Mother, I could have shouted, but somehow I dared not hurt little Brown's feelings."

This was the beginning of a rapidly formed friendship between the two boys. When Master Johnny went birds'-nesting in the woods or rabbit-trapping on the moorland, Toby followed, his devoted shadow. The odd little creature conceived a huge affection for the young master from "t' Hall," and would have done anything on earth to win his careless word of approval.

"I say, Toby, I want you this afternoon!" It was a frosty, winter morning, and Johnny's footsteps rang out on the hard, flinty ground as he tore along to the rectory, where he learnt his lessons with the rector's boys. In all weathers Toby Brown was safe to be on the watch for a look or a word before trudging off to school, so was ready with his "All right, Master Johnny," guessing correctly that some sliding was in prospect.

Afternoon came, and the two boys met. "Toby, my mother has ordered the keepers to watch that I don't go on the pond until to-morrow, when she thinks the ice will be thicker. Now, do you think I'm going to lose all this jolly frosty day? No! Well, then, Toby, do you know any pond where we could go and have a good time by ourselves?"

Yes, Toby did. It was not long, therefore, before the two were having a glorious spin over the smooth ice of a pond, which they had all to themselves, unfortunately; for when Master Johnny went headlong into a hole, broken in the ice for the water-fowl, there was not a soul near to aid him but Toby. For a moment that individual was puzzled. This sort of accident was beyond his ken. Johnny, who was up to his neck in water, screamed lustily, being in a sore plight.

"You keep still, master. You be a-breakin' the ice wusser. Jes' you wait." Toby had torn off his jacket, and, lying flat down, he flung it with all his might, Johnny just managing to clutch at the cuff of a sleeve.

Then Toby set to work manfully, and, between his tugging and Johnny's efforts, the latter was landed at last upon the cracking ice, and the boys stood up, looking into each other's faces with quivering lips.

"You put on my jacket, Master Johnny, it'll keep 'ee warm." But that it could not, for when Johnny, a dripping spectacle, presented himself at The Hall door, the icicles were forming upon him, while behind him shivered Toby, with chattering teeth, and in his shirt-sleeves. The result, of course, was a severe illness for each—Toby turning up in hospital once again.

But all that seems a long time ago. The little lads are now men—both defenders of their country. When Johnny at The Hall passed and went into the army, what was there left for Mrs. Brown's boy to do but slip off from the village and enlist in the young officer's regiment, just as it started for India.

"Why should you lament and cry aloud thus?" said Johnny's mother, as she tried to quiet Mrs. Brown's clamorous grief. "Is your son more to you than mine to me, think you? If I can spare my only boy to go out and fight for England, cannot you also give yours to the same good cause?"

After that there was a lull, and Mrs. Brown went about telling with pride how her Toby and Master Johnny had gone off "to Indy to look after things in that there Burmah."

Nobody was surprised, however, to hear, in the first letter home, that Toby was already in hospital—a touch of jungle fever. "Lor!" said his mother, "if so be as there's a 'orsepital, our Toby's safe to get inside o' it, he is."

The next news came in the daily papers. There had been a skirmish, destructive to the enemy. Special mention was made in the despatches of two heroes; and Johnny's mother and sister wept and smiled by turns on reading the General's praise of the young officer's brave pursuit of the natives, who, finding but one pale face at their heels, would have cut him down save for gallant Private Tobias Brown, who came up in time to hack, hew, and disperse the black cowards. It was a deed of heroism common enough—we can say so with proudly-beating hearts—in our British Army, where courage to recklessness is every man's heritage.

"And be they both in 'orsepital?" asked Mrs. Brown, when her tears were dried.

"Both," said Johnny's mother, "and doing wonderfully, thank God!"

By-and-by—a long by-and-by it seemed, indeed, a couple of years slipped past—there came the best news of all.

"We're coming home on leave, mother dear, Brown and myself. Of course, if I come, he follows—for he is called my shadow," wrote Captain John.

When the time drew near for the arrival of the two brave villagers, their native place was *en fete* to receive them with welcoming cheers and triumphal arches, besides a round of festivities and flattering speeches calculated to render anyone but British warriors light-headed. Perhaps, of everybody, Toby's mother felt the most elated, for it was her own opinion—and the secret one of many a sweet-faced maiden—that Mrs. Brown's boy completely "took the shine out o' the young captain," seeing that Toby marched about the village loftily in a smart uniform, which compared favourably with the squire's rough grey tweed suit.

"And when are you coming to settle down, dear boy?" asked the captain's mother, smoothing her son's short, curly hair fondly. "We want you here at The Hall to fill your father's place, empty so long."

"Time enough for that, mother. I must see a little more life and fighting before I'm content to be a country squire. But don't worry; the day will come in good time."

"Yes," said Edie; "and I suppose that day will see Toby Brown at your heels to settle down as porter at the hospital."

"Of course, of course," answered her brother, in all simplicity; and doubtless it will be so.

### The Management of Children.

### III.—MINOR AILMENTS.

Every nurse who has had charge of children must have been struck at some time with the rapid way in which they fail or pick up health. It needs very little to kill an infant; an improper meal brings on fatal convulsions, or a head flannel gets over the infant's mouth at night and suffocates it, and baby is dead. The high rate of infantile mortality is one of the disgraces of this century, when so much is being done for the preservation of life. Care in small things and unceasing watchfulness are necessary on the part of a children's nurse; it is impossible that anything connected with the nursing of infants can be a trifle, and it is here that success lies for the good nurse. If the children fail rapidly they also pick up rapidly. An infant at death's door, dying of neglect and improper food apparently, and a mass of sores, is brought to the hospital. Baths and cleanliness, an equal temperature, regular wholesome food, and constant care, and baby is soon fat and well, and crowing contentedly in its cradle. It is because of this mercurial temperament in children that we do not speak of slight illnesses, but rather of minor ailments. No illness is slight which attacks a child, and a nurse should know how to care for the victim of a cold or an attack of diarrhœa just as much as how to attend to a tracheotomy or meningitis case. Miss Wood gives a simple example of the difference in the manner in which a clever and stupid nurse start in feeding a baby. The last will settle the baby on her lap with hands and feet free, and something near diverting its attention, and then say it is a troublesome child to feed. The clever nurse will wrap the child in a blanket, which will confine hands and feet, and not allow interruption till the food is finished. It is in small points like these that experience tells, though there are some women who by no amount of teaching ever learn how to manage children, and these women, of course, should never try to nurse sick children. "A Mother" gives excellent directions for the preparing and administering of castor oil to children, saying, in conclusion, that a child should lie down and try and sleep after the dose, so avoiding any feeling of nausea. All these small points tell in the management of children, and help to keep the minor ailments from becoming major ones.

Colds, croup, whooping-cough, thrush, convulsions, measles, and chicken-pox, are all counted amongst the minor ailments, and all need careful watching and treatment lest they become serious. In nursing private cases of these common complaints it is very hard to prevent parents and friends from experimenting with popular remedies. A nurse was watching a small child through severe whooping-cough once, and everyone who came to see the child, or to see its mother, advised some safe and certain cure. The nurse listened respectfully and made notes; but of course did not depart from the routine laid down by the doctor. At the end of the case she showed the mother her notes; they consisted of 42 different remedies, from holding the child head downwards in a freshly dug hole in the earth "so that it may breathe the fresh smell," down to the simple prescription of constant hot baths. A nurse who has charge of a child through minor ailments should not restrict her attentions to the cough or sore throat only; it is necessary also to build up the child's general health, and it is well to train it in regular habits. Miss Wood gives some laws of life, which we will quote in taking leave of "A Handbook for the Nursing of Sick Children" and "The Management of Children." The laws of life are these: Fresh air; sunshine and light; suitable food; cleanliness; warmth; exercise; rest and recreation; order and regularity.

### British Nurses' Association.

### A NEW DEPARTURE.

It looks as if the haughty were conscious of having mightily fallen. At the start all lay governors of hospitals were warned off as know-nothings and impediments to progress. Now the scene has changed. The British Nurses' Association, or rather its guiding spirits, have at last taken a step which they should have done before canvassing matrons and nurses to subscribe their names and money to their ideal but chimerical project. They now condescend to appeal to the constituted authorities of hospitals and nurse training-schools to induce them, if possible, to appoint representatives to their registration council, being "most anxious by every means in their power to carry the hospital authorities with them." This is an entirely new departure, but we have good reason to know that it comes too late to be of any avail. The circular addressed to the various hospitals is couched in language with which we are only too familiar, commenting on the dangers of employing nurses and midwives unworthy of trust, as if anyone in their senses would do anything of the kind, while hospitals and all respectable nursing institutions, as the public know well, have the power in their own hands to prevent it. The alliance which would be brought about by a common register for nurses and midwives would be most unfortunate, and the suggestion of this juxtaposition on equal terms must sorely puzzle the wisdom of the committees to whom the circular is addressed. The business of a midwife is of a purely commercial character, followed by women after three or six months' experience of the lying-in charity and on passing a satisfactory examination; while that of a sick nurse necessitates a long (three years) probation in a very different field, her aspirations, from the very nature of her calling, being as much benevolent as mercantile in their scope. The great aim of hospital managers is to maintain a high moral tone among their nurses; on this almost everything else depends. There is no greater mistake than to delude them with the flattering assumption that nursing is a learned profession which requires from its votaries some diploma or badge, altogether apart from the credentials they receive from the authorities of their alma mater, who are best acquainted with the nurse's character, length of experience and capabilities. In the face of the protest from the leading nurse-training schools in London against registration on the lines proposed by the British Nurses' Association, we shall watch with curiosity the decision of provincial hospitals with reference to the question. No doubt hospital managers will remember that they are only now invited to come and help the British Nurses' Association at the eleventh hour, when the project of the charter has not been proceeded with, and that although promised frequently no definite scheme of registration or indeed any other practical work has been yet forthcoming. As we go to press we hear the authorities of St. Bartholomew's Hospital have promptly declined to entertain this new scheme of registration, and no doubt provincial hospital authorities will be careful not to commit themselves to any impractical project of a similar character.

### Lectures, Etc.

Dr. Alice Vickery will lecture to women only at the Farm House, Harrow-street, opposite St. George's Church, Borough, on November 29 and December 13.

At the Working Men's College, Great Ormond-street, on November 23, at eight o'clock, George Carfrae, M.D., on "Homœopathy." On November 30, T. Robinson, M.D., on "Antipathies." On December 7 and 14, George Cunningham, F.D.S., on "Our Teeth," with limelight illustrations. All free.

The Sunday afternoon oratorios at St. Nicholas' Cole Abbey were recommenced on Sunday, October 20. There are organ recitals in the same church every Tuesday at 1 p.m.

### Death in Our Ranks.

IN the early morning of Nov. 2nd, at the Red House ("Bromhead Memorial"), Lincoln, died Nurse Margaret Marris, from typhoid fever contracted during her attendance on a patient. Nurse Margaret was nearly 29 years of age, and had been a member of the Lincoln Nurses' Institution for five years, where she was greatly respected by her fellow nurses, who deeply regret her death.

NURSE MCMILLAN, of the Ryde Infirmary, died last week from an overdose of morphia. The deceased, who was only 23 years of age, had been suffering from sleeplessness, and on the Sunday morning injected an overdose of morphia, and in spite of all efforts to save her, died that evening. The funeral was largely attended, Miss Fitzpatrick (matron) and the nurses being present. This sad death is a serious lesson to all nurses who are tempted to use their opportunities to indulge in drugs to secure sleep.

### Notices.

GREAT YARMOUTH HOSPITAL.—The various applicants for the post of matron at this hospital can have their testimonials returned by sending a stamped addressed envelope to the secretary before Nov. 26. After this date all testimonials will be destroyed.

EXHIBITION OF NURSING UNIFORMS.—The exhibition of nursing uniforms will be open from Dec. 9 to 14. All nurses in uniform will be admitted free. Further particulars next week. All the best hospitals are now represented, save Middlesex, Liverpool Royal, Manchester Royal, and the Naval Nursing Sisters. Are there no friends of these institutions who will come forward in their interests and forward us the dolls before next Saturday? We have had the offer of the loan of the original badge for the National Aid Association's sisters, worked by H.R.H. the Princess of Wales, for exhibition. If any matrons, sisters, or nurses have other small objects of interest they would like to exhibit, will they please write at once, and give particulars to the Editor of THE HOSPITAL, The Lodge, Porchester-square, London, W., writing "Exhibition" in the top left-hand corner of the envelope.

### Appointments.

CENTRAL LONDON OPHTHALMIC.—Miss Marian Hardwick has been appointed matron of this hospital. She trained under Sister Dora at Walsall, from whom she has a testimonial, and then went to King's for three months. Miss Hardwick has had a very varied experience, including work at Leicester Infirmary, district work at Cambridge, the charge of Clevedon Cottage Hospital, a year at Winchester, a temporary matron's duty at Rugeley, Bath, Birkenhead, Grimsby, Wallingford, Basingstoke, and Teddington. She ought, therefore, to be able to give every satisfaction to the committee who have elected her to a permanent post.

### Notes and Queries.

### Queries.

(24) *Medical Dictionary.*—Is there one published small enough for the pocket? *Nurse Nora.*

*Nursing at the Cape.*—Can anyone give information as to whom to apply for particulars? *Helen.*

### Answers.

(24) *Medical Dictionary.*—The smallest we know is Lewis's Medical Vocabulary, published by H. K. Lewis, of Gower-street, price 3s.

*Brixton Nursing Lectures.*—Miss G. should apply for information to the Secretary of the National Health Society, Berners-street, W.

*Miss Willcox.*—Your name has been added to the list.

*M. B.*—Had you sent us news of your appointment at the time we would gladly have inserted it, but now the news is stale and of no interest. We willingly insert news sent, but it is no use to be angry with us for not knowing every hospital appointment throughout the United Kingdom. Those who want their appointments to appear in our pages should take the trouble to write and send particulars.

*Helen.*—There is no charge; we insert all queries and answers free.

*Nursing at the Cape.*—Seven nurses have gone to Port Elizabeth Hospital, Cape Colony, from Glasgow Royal Infirmary; perhaps the authorities at Glasgow could give information. Direct application might also be made to the matron at Kimberley.

### Presentations.

MR. WILLIAM LEES, M.R.C.S., for upwards of five years house surgeon to the Chester General Infirmary, has been presented by the nursing staff of the institution with two handsome cases of surgical instruments, and the same staff has also presented Mr. Alfred Mann, M.B., C.M. Edin. (who has retired from the office of visiting surgeon), with a similar case of instruments.

### Vacancies.

THE following vacancies are announced:

### Matrons.

Dr. Walter's Hospital, Manchester, £36.

### Sister.

West London Mission.

### Nurses.

Amersham Union, £25.
Bath United Hospital, £25.
Bedfordshire Institute, £26.
Belfast Nurses' Home.
Blackheath Institute, £20.
Bromley Infectious Hospital, £18.
Cardiff Infirmary.
Cheltenham Institution.
Children's Hospital, Paddington.
Croydon Union (Male), £30.
Eastbourne Nursing Institution.
Frome Nurses' Home.
Glasgow Maternity Hospital.
Great Yarmouth Hospital (night), £22.
Hertford Infirmary, £20.
H. M. Hospital, Stepney, £25.
Jenny Lind Infirmary, £20.
Kent Nursing Institution (five), £30.
King's Cross Hospital, Dundee, £24.
Kingston Home, Hull.
Leicester Institution, £25.
London and Brighton Nurses' Association (three).
Longton Cottage Hospital.
Middlesbrough Fever Hospital.
Newcastle City Hospital, £25.
Newport Nursing Institute.
Northampton Infirmary, £20.
Nursing Institution, 96, 97, 98, Wimpole-street, London, W., Mr. Wilson has several vacancies for thoroughly trained nurses.
Royal Albert Hospital, Devonport (two), £25.
Scarborough Institute.
South-Eastern Fever Hospital, £30.
Southport Infirmary, £25.
South-Western Fever Hospital (several), £30.
St. John's Institution, Southport (several), £25.
St. Leonard's Home.
St. Saviour's Infirmary, Dulwich, £20.
Swansea Institute.
Truro Infirmary.
West Bromwich Hospital, £22.
West Herts. Infirmary (night).
West London Hospital, £24.
Whitechapel Infirmary, £20.
Windsor Infirmary (night), £20.
Worcester Infirmary, £25.
York Nurses' Home.

### District Nurses.

District Home, Bolton.
Elford House, Coventry, £20.

### Probationers.

Barnet Cottage Hospital.
Batley Hospital.
Boston Hospital.
Canterbury Hospital.
Dawlish Cottage Hospital.
Great Yarmouth Hospital.
H. M. Hospital, Stepney.
Lowestoft Hospital.
South-Western Fever Hospital.
Truro Infirmary.

### Amusements and Relaxation.

**Third Quarterly Word Competition Commenced October 5th, 1889, ends December 28th, 1889.**

Three Prizes of 15s., 10s., 5s., will be given for the largest number of words derived from the words set for dissection.

Proper names, abbreviations, foreign words, words of less than four letters, and repetitions are barred; plurals, and past and present participles of verbs, are allowed. Nuttall's *Standard* dictionary only to be used.

N.B.—Word dissections must be sent in WEEKLY, arranged alphabetically, with correct total affixed.

The word for dissection for this, the *Eighth* week of the quarter, being "ELEPHANTS."

### Results of Sixth Week.

| Names. | Nov. 14th. | Totals. | Names. | Nov. 14th. | Totals. |
|---|---|---|---|---|---|
| Winifred | — | 672 | Sunshine | 7 | 814 |
| Adelaide | — | 646 | Electrician | 6 | 944 |
| Nettie Vance | 6 | 757 | H. C. | — | 562 |
| Amos | — | 596 | Reldas | 7 | 976 |
| F. W. P. H. | 7 | 959 | Rover | — | 776 |
| Reyot | — | 369 | Qu'appelle | 6 | 796 |
| Ellice | 6 | 998 | Beryl | — | 188 |
| A. S. K. | — | 275 | Lightowlers | 6 | 1032 |
| Neptune | 7 | 961 | Hercules | — | 12 |
| Ecila | 7 | 1048 | Jenny Wren | 7 | 649 |
| Tinie | — | 17 | Night Watch | — | 346 |
| Amicus | 4 | 946 | Oakenrod | 6 | 1031 |
| Juvenis | — | 539 | Embryo | — | 385 |
| Jumps | 7 | 1054 | Gypsye | 5 | 876 |
| Sister | — | 438 | Leo | 6 | 676 |
| M. W. | 7 | 999 | Pearl | — | 299 |
| Esperance | 7 | 1003 | Africando | 4 | 171 |

### Notice to Correspondents.

N.B.—All letters referring to this page which do not arrive at 139 and 140, Salisbury-court, London, E.C., by the *first post on Thursdays*, and are not addressed PRIZE EDITOR, will in future be disqualified and disregarded.

